# Supplementary material for: Impacts of Psychopharmaceuticals on the Neurodevelopment of Aquatic Wildlife: A Call for Increased Knowledge Exchange across Disciplines to Highlight Implications for Human Health
Source: Int J Environ Res Public Health. 2021 May 12;18(10):5094. doi: 10.3390/ijerph18105094 (PMC8151291; doi:10.3390/ijerph18105094)
Supplement: Supplementary file 1 [file ijerph-18-05094-s001.zip › ijerph-1165198-supplementary.pdf]

SUPPLEMENTAL MATERIAL

SUPPLEMENTAL METHODS

Python script

```
## Takes in list of journals and citations and outputs weighted network links
## Written for "Contaminants in Waterways" technical analysis and data visualization

import csv

# Global variables and data structures
journals = {}
categories = {}
citations = {}
total_journals = 0

# Read in list of distinct journals and initialize their category to "other"
def getJournals():
    global journals,total_journals
    with open('network/distinctJournals.csv', 'r') as csvfile:
        data = csv.reader(csvfile)
        for d in data:
            journals[d[0].lower()] = "other"
            total_journals +=1

# Read in categories of interest which must appear in order of priority
def getCategories():
    global categories
    with open('categories/categoryList.csv', 'r') as csvfile:
        data = csv.DictReader(csvfile)
```

```
        for d in data:
            categories[d["category"]] = {"count":0,"area":d["subjectArea"],"name":d["fullName"]}

# Iterate over each category of interest and categorize all journals
def categorizeJournals():
    global categories,journals,total_journals
    categorized = 0
    for category in categories.keys():
        with open("categories/" + str(category)+'.csv', 'r') as csvfile:
            data = csv.DictReader(csvfile, delimiter=";")
            for d in data:
                if d["Title"].lower() in journals.keys() and journals[d["Title"].lower()] == "other":
                    journals[d["Title"].lower()] = category
                    categories[category]["count"] += 1
                    categorized += 1

    # Journals that remain uncategorized are grouped as "Other"
    categories["other"] = {"count":total_journals-categorized,"area":"Other","name":"Journals that do not fall under any category of interest"}

# Categorize citations according to their journals' categories
def categorizeCitations():
    global journals,citations
    with open('network/citations.csv', 'r') as csvfile:
        data = csv.DictReader(csvfile)

        # If A cited B, then A is the citer and B cited, arrow from A to B
        for d in data:
            citer = journals[d["journal"].lower()]
            cited = journals[d["source"].lower()]
```

```
            # Keep track of citation weights
            if (citer,cited) in citations.keys():
                citations[(citer,cited)] += 1
            else:
                citations[(citer,cited)] = 1

# Format element data as csv for Kumu data visualization
def outputElements():
    global categories
    with open('kumu/KumuElements.csv', 'w') as csvfile:
        csvfile.write("Label,Journals,Type,Description\n")
        for category in categories.keys():
            c = categories[category]
            output = [category,c["count"],c["area"],str(c["area"])+": "+str(c["name"])]

            # Kumu element format is Label,Journals,Type,Description
            csvfile.write('"{}",{},"{}","{}"\n'.format(category,c["count"],c["area"],str(c["area"])+": "+str(c["name"])))

# Format link data as csv for Kumu data visualization
def outputLinks():
    global citations
    with open('kumu/KumuLinks.csv', 'w') as csvfile:
        for citation in citations.keys():

            # Kumu link format is csv From,To,Weight
            csvfile.write(str(citation[0]) + "," + str(citation[1]) + "," + str(citations[citation])+"\n")

# Driver code
getJournals()
getCategories()
categorizeJournals()
```

categorizeCitations()

outputElements()

outputLinks()
